# Quantitative Changes in Muscular and Capillary Oxygen Desaturation Measured by Optical Sensors during Continuous Positive Airway Pressure Titration for Obstructive Sleep Apnea

**DOI:** 10.3390/bios12010003

**Published:** 2021-12-21

**Authors:** Zhongxing Zhang, Ming Qi, Gordana Hügli, Ramin Khatami

**Affiliations:** 1 Center for Sleep Medicine, Sleep Research and Epileptology, Clinic Barmelweid AG, 5017 Barmelweid, Switzerland; ming.qi@barmelweid.ch (M.Q.); gordana.huegli@barmelweid.ch (G.H.); ramin.khatami@barmelweid.ch (R.K.); 2 Barmelweid Academy, Clinic Barmelweid AG, 5017 Barmelweid, Switzerland; 3 Department of Neurology, Inselspital, Bern University Hospital, University of Bern, 3010 Bern, Switzerland

**Keywords:** obstructive sleep apnea, continuous positive airway pressure therapy, near-infrared spectroscopy, oxygen desaturation, arm, pulse oximeter, wearable

## Abstract

Obstructive sleep apnea (OSA) is a common sleep disorder, and continuous positive airway pressure (CPAP) is the most effective treatment. Poor adherence is one of the major challenges in CPAP therapy. The recent boom of wearable optical sensors measuring oxygen saturation makes at-home multiple-night CPAP titrations possible, which may essentially improve the adherence of CPAP therapy by optimizing its pressure in a real-life setting economically. We tested whether the oxygen desaturations (ODs) measured in the arm muscle (arm_OD) by gold-standard frequency-domain multi-distance near-infrared spectroscopy (FDMD-NIRS) change quantitatively with titrated CPAP pressures in OSA patients together with polysomnography. We found that the arm_OD (2.08 ± 1.23%, mean ± standard deviation) was significantly smaller (*p*-value < 0.0001) than the fingertip OD (finger_OD) (4.46 ± 2.37%) measured by a polysomnography pulse oximeter. Linear mixed-effects models suggested that CPAP pressure was a significant predictor for finger_OD but not for arm_OD. Since FDMD-NIRS measures a mixture of arterial and venous OD, whereas a fingertip pulse oximeter measures arterial OD, our results of no association between arm_OD and finger_OD indicate that the arm_OD mainly represented venous desaturation. Arm_OD measured by optical sensors used for wearables may not be a suitable indicator of the CPAP titration effectiveness.

## 1. Introduction

Obstructive sleep apnea (OSA) is the most prevalent respiratory sleep disorder, occurring in 9–38% of the general population [1], and it is a high-risk factor for many diseases, such as cardio-/cerebrovascular diseases [2,3], periodontal disease [4] and type 2 diabetes [5]. Although different treatments (e.g., oral appliances, such as mandibular advancement devices, or other treatments, such as positional therapy, uvulopalatopharyngoplasty and hypoglossal nerve stimulation) [6] are available, continuous positive airway pressure (CPAP) or automatic positive airway pressure (APAP) therapy is currently the most effective treatment for OSA [7,8]. However, there are still many challenges in OSA diagnosis and treatment, particularly simple low-cost new diagnostic technologies that can be easily used by the patients for home recordings are urgently needed [9,10]. This urgency is due to observations that: (1) the majority of suspected OSA patients remain undiagnosed in many countries due to the lack of sleep laboratories/specialists, the little diagnostic value of daytime sleepiness for most people and the high cost of in-lab polysomnography (PSG) diagnosis [11,12,13]; and (2) long-term home recordings employing these easy-to-use technologies will be helpful to longitudinally monitor the CPAP treatment effects and adherence or even to select patients who will benefit most from treatment (i.e., precision or personalized medicine) [9,14].

Recently, low-cost wearable devices, such as smartwatches (e.g., the new products from Fitbit [15], Garmin [16], Huami [17] and Huawei [18]) and armbands (e.g., Humon [19,20], Moxy [21,22], Artinis [23] and Biofourmis [24]), have implemented the function of measuring peripheral capillary (SpO2) or muscle tissue (StO2) oxygen saturation at the arm or wrist using near-infrared light, making them possible alternatives for at-home OSA measurement. Unlike the in-lab PSG fingertip pulse oximetry, in which the detector measures the light transmitted through the fingertip (i.e., transmission photoplethysmography (T-PPG)), these wearable devices assess the SpO2 or StO2 by measuring the changes in the backscattering light from the wrist or arm based on the modified Beer–Lambert law (MBLL) [25,26,27,28]. That is, their light sources are usually two or more near-infrared light wavelengths mainly absorbed by oxygenated hemoglobin (HbO2) and deoxygenated hemoglobin (HHb) in biological tissues, and detectors are placed on the same side of the measured tissues because the wrist and arm are too thick to be penetrated by light. While wrist wearable devices mainly use reflectance photoplethysmography (R-PPG) or pulse oximetry [28,29,30], armbands are mainly based on simple continuous-wave near-infrared spectroscopy (CW-NIRS) methods [20,22].

Measuring OSA by these low-cost wearable devices is unfortunately still not viable because none of them has been licensed or certified as a medical diagnostic device by the United States Food and Drug Administration or CE marking [31]. In our recent study [32], we compared event-by-event oxygen desaturation (OD) at the fingertip (finger_OD) measured by gold-standard in-lab PSG fingertip T-PPG with the OD at arm (arm_OD) measured by gold-standard frequency-domain multi-distance near-infrared spectroscopy (FDMD-NIRS) in OSA patients during naïve sleep and during CPAP titrations. Our Bland–Altman plots yielded poor agreement between finger_OD and arm_OD in sleep hypopneas, suggesting that the absolute value of arm_OD is not suitable to define sleep hypopneas according to the standard clinical criterion of ≥3% OD drop. It could lead to large false-negative results in measuring OSA events, thus underestimating the Apnea–Hypopnea index (AHI) compared to in-lab PSG.

However, whether the changes in arm_OD can indicate the effectiveness of CPAP titration remains unknown. The CPAP/APAP machine itself can measure the AHI, although its accuracy has been criticized [33,34,35]. Two major challenges in CPAP/APAP treatment are poor adherence to treatment [36] and persisting hypoxemia and daytime syndromes despite therapy in some patients, for whom oxygen supplementation may be needed [9]. Logically, ‘A does not equal to B’ does not necessarily mean that ‘changes in A are not associated with changes in B’. For example, obesity is a risk factor for OSA. A high BMI cannot be used to replace a high AHI to define OSA, but the decrease in the BMI is associated with a decrease in the AHI [37]. If the changes in arm_OD can be associated with changes in CPAP/APAP pressures, then measuring changes in arm_OD with a wearable device at home could be helpful to better understand those patients, e.g., to identify subgroups with/without improvement in OD when CPAP/APAP pressure changes. It could also make at-home multiple-night CPAP titrations possible, i.e., to find the optimal CPAP pressure that can at best restore the respiratory events and arm_OD, which may essentially improve the adherence of CPAP therapy by optimizing its pressures in a real-life setting economically.

Therefore, in this study, we quantified the dynamic changes in arm_OD in OSA events under titrated CPAP pressures using regression analysis. We used the same dataset that has been recently published in [32], considering its major advantages, which include: (1) the CPAP pressures were well-controlled stepwise and increased hourly during titration; (2) the simultaneously measured finger_OD can serve as a control for CPAP titration effectiveness, i.e., an effective titration procedure should restore stepwise the fingertip SpO2 desaturation; (3) the comparisons between finger_OD (i.e., arterial desaturation) and arm_OD (i.e., the mixture of arterial and venous desaturation [38,39]) may provide new insights into the peripheral desaturation in response to OSA events during CPAP therapy. We hypothesized that the changes in arm_OD may be associated with changes in CPAP pressures, considering that our stepwise incremental CPAP titration gradually opened the upper airway to increase oxygen supply.

## 2. Materials and Methods

### 2.1. Study Design

Thirty newly diagnosed OSA patients (age (mean ± standard deviation, SD): 54.2 ± 13.8 years, interquartile range (IQR) 42–65 years; male: *n* = 27; body mass index (BMI): 35.9 ± 7.5 kg/m^2^, IQR 31.8–42.0 kg/m^2^; AHI: 53.4 ± 24.7 per hour, IQR 32–71 per hour) participated in this study. Patients with unstable coronary or cerebral artery disease, severe arterial hypertension or hypotension, respiratory diseases or a history of a sleep-related accident were excluded from this study. This study was approved by the local ethical commission of Northwest Switzerland and was in compliance with the Declaration of Helsinki. Written informed consent was obtained from all patients prior to their participation.

Patients underwent incremental stepwise CPAP (AirSense™10, ResMed) titration combined with video-PSG and FDMD-NIRS recordings in one nocturnal sleep episode. This sleep episode consisted of 1 h of baseline sleep without CPAP followed by incremental stepwise titration of 1 cmH2O pressure per hour starting from 5–8 cmH2O depending on the individuals. Video-PSG (Embla RemLogic, Natus Medical Incorporated, Tonawanda, NY, USA) is a comprehensive recording of physiological signals during sleep, including electroencephalography, electrooculogram, electromyogram, electrocardiogram, breathing functions, heart rate (HR), fingertip SpO2 and movement during sleep. Two experienced sleep technologists independently scored the sleep stages, respiratory events (sleep apneas and hypopneas) and motion artifacts in 30 s epochs according to the 2017 American Academy of Sleep Medicine manual [40]. The discrepancy between these two technologists was resolved by discussion or recommendation by an experienced neurophysiologist.

FDMD-NIRS (Imagent, ISS, Champaign, IL, USA) measurements were conducted over the middle of the left biceps muscle. Imagent is currently the only commercial benchtop FDMD-NIRS device [41,42,43] and has been CE-approved for research. The robustness, precision and accuracy of measuring hemodynamics of the Imagent system have been well validated in different physical blood-lipid models [42,44,45] and in vivo studies [46,47,48,49]. It has been used as a gold-standard reference measurement of StO2 for the validations or calibrations of wearable CW-NIRS armbands [20] and portable CW-NIRS oximeters including those that have received FDA clearance [45,50]. Its light emitters, four laser diodes at 690 nm wavelength and four laser diodes at 830 nm wavelength are coupled into four light sources and are high-frequency-modulated at 110 MHz. The light can penetrate into the measured tissues with a depth of several centimeters when the four light sources are aligned and placed at 2 cm, 2.5 cm, 3 cm and 3.5 cm from an optical fiber bundle connected to the photomultiplier tube detector. The sampling rate of FDMD-NIRS recording was set as 5.2 Hz. The Imagent system was calibrated on an optical phantom block to exclude the uncertainty of measurements due to machine errors before each recording. The raw measured NIRS data were subjected to a low-pass (<0.08 Hz) zero-phase filter designed using a Hanning window to remove the physiological noises including heart rate, respiratory noise and spontaneous slow hemodynamic oscillations [32,51,52]. The filtered data were then smoothed using the robust locally weighted scatter plot smoothing method [51,53].

### 2.2. Statistical Analysis

**Figure 1:**
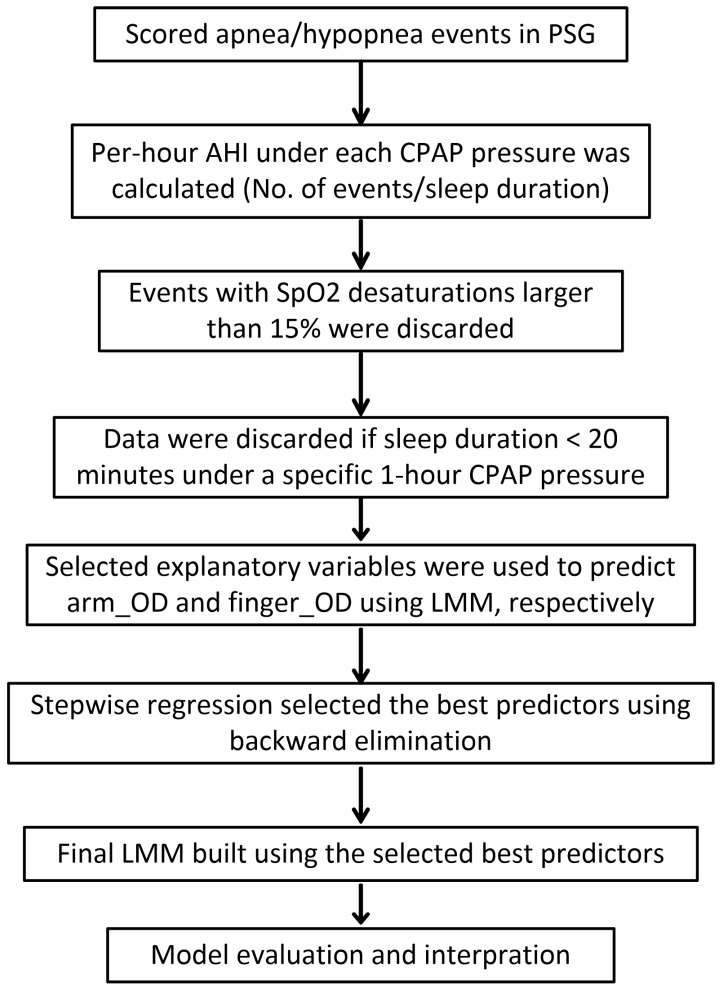
Data analysis procedure.

The data analysis procedure is shown in Figure 1. After a standard PSG scoring, per-hour AHI under each CPAP pressure was calculated, i.e., the number of events was divided by the sleep duration under each CPAP pressure per hour in the titration protocol. Obstructive apneas (*n* = 29) and hypopneas (*n* = 31) were excluded from analysis if their SpO2 desaturations were greater than 15% to exclude outliers and potentially unreliable measurements caused by instrument errors [30,32]. In each patient, all the events under a specific CPAP pressure were also excluded if the corresponding sleep duration under that pressure was shorter than 20 min to exclude the unreliable calculation of the per-hour AHI. For example, if the sleep duration under some CPAP pressure was just a few minutes while the patient had a number of apneas/hypopneas, the calculated per-hour AHI could be extremely large, but its value was unreliable due to short sleep duration. Then linear mixed-effects model (LMM) with a random intercept by patients was used to predict the arm_OD and finger_OD caused by the respiratory events, respectively. Explanatory variables were demographic variables (i.e., age, sex, BMI, AHI of the diagnostic night measured by PSG) and parameters that can be measured by CPAP machine or wearable devices, i.e., types of respiratory events, durations of event, sleep stages, mean HR during the events, per-hour AHI under each pressure and CPAP pressures. Stepwise regression using backward elimination was performed to automatically select the best predictors. Then the final LMM models were built using these best predictors. We reported both the conditional *R*^2^ [54] and *Ω*^2^ (i.e., Xu’s *R*^2^ calculated as 1—variance of residual/variance of response) [55] to assess the goodness of fit of our final selected models.

Data were expressed as the mean ± SD unless otherwise indicated. The pre-processing of FDMD-NIRS signals was carried out in MATLAB (The MathWorks, Inc., Natick, MA, USA). All statistical analyses were performed using R (version 3.2.4). The LMM models were created using the R package *lme4* (function *lmer*) and stepwise regressions were performed using the R package *lmerTest* (function *step*).

## 3. Results

**Figure 2:**
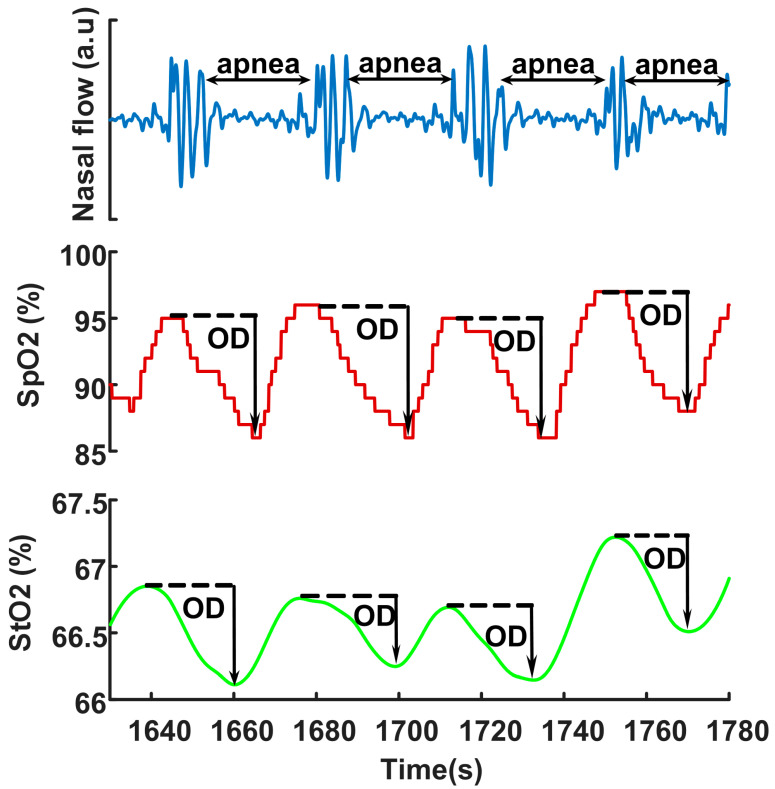
Typical fingertip SpO2 desaturation and arm StO2 desaturation during apneas. The arrow indicates the degree of oxygen desaturation (OD). SpO2 is measured at PSG fingertip by transmission photoplethysmography, and StO2 is measured at biceps muscle by FDMD-NIRS.

In total, 505 obstructive apnea and 2185 hypopnea events were analyzed. The median of the number of events acquired from our patients was 75 with an IQR between 59 and 110. Figure 2 illustrates typical changes in fingertip SpO2 and arm StO2 desaturations in OSA events. ODs triggered by sleep apneas occurred in both SpO2 and StO2, although the baseline StO2 values (mostly between 60 and 70%) were smaller than SpO2 (mostly above 90%) because StO2 was from both venous and arterial blood. The mean arm_OD (2.08 ± 1.23%) was significantly smaller (paired *t*-test, *p*-value < 0.0001) than the mean finger_OD (4.46 ± 2.37%). There was no correlation between the degrees of arm_OD and finger_OD, indicated by the Pearson’s correlation coefficient of 0.08 (*p*-value < 0.0001).

The results of the final LMMs predicting arm_OD and finger_OD selected by stepwise regressions are shown in Table 1 and Table 2, respectively. The conditional *R*^2^ and *Ω*^2^ of the model for arm_OD were 0.66 and 0.69, respectively. These two values of the model for finger_OD were 0.51 and 0.49, respectively. CPAP pressure was a significant predictor for the finger_OD (i.e., the increase of one unit pressure was associated with 0.12% less decrease in fingertip oxygen desaturation) but not for arm_OD.

It could be possible that the normalized changes rather than the raw values of arm_OD were associated with CPAP pressures since the StO2 baseline level (68.6 ± 6.4%) before the desaturations was obviously much smaller than that of SpO2 (usually above 90%). We, therefore, normalized the arm_OD to its baseline and repeated the LMM analysis. The CPAP pressure was still automatically excluded from the final selected model in stepwise regression, in which the final selected predictors were the same as shown in Table 1, i.e., the duration of events (estimated coefficient 0.033, *p*-value < 0.0001), mean HR within events (estimated coefficient −0.03, *p*-value < 0.0001) and per-hour AHI (estimated coefficient 0.0028, *p*-value = 0.00058).

## 4. Discussion

In this study, we tested whether oxygen desaturations measured in the arm muscle change with CPAP pressures during CPAP titration in patients with OSA. In contrast to our recent study testing the agreement between arm and fingertip oxygen desaturations in sleep hypopneas using Bland–Altman plots [32], here we used linear regression (i.e., LMM) to study the association between CPAP pressures and oxygen desaturations at arm and fingertip. Contrary to our hypothesis, we only found association between CPAP pressures and the oxygen desaturations at the fingertip but not in the arm muscle. Our negative results suggest that muscular oxygen desaturation may be not a suitable indicator of the effectiveness of CPAP titration. Thus, the usefulness of wearable devices measuring arm StO2 in CPAP therapy is questionable.

Only fingertip SpO2 but not the arm StO2 reflects the reduction of desaturations during CPAP titration, probably because venous blood contributing to StO2 reduces the sensitivity of StO2 in response to CPAP pressures compared to SpO2. The NIRS StO2 is the proportion of HbO2 in the measured biological tissues including arterial, capillary and venous compartments. It can be expressed as:

StO2 = *a* × SaO2 + *b* × SvO2 (1)

where SaO2 and SvO2 are the arterial and venous oxygen saturation [38,39]. SaO2 is approximately equal to SpO2 as they are both arterial oxygen saturation (usually close to 100%). SvO2 is usually 65–75% [56]. The ratio of coefficients *a*/*b* is the arterial-to-venous volume ratio (AVR), and *a* + *b* = 1. Most commercially available NIRS oximeters including the FDA-certificated medical devices usually fix the AVR as either 25%/75% or 30%/70% [57,58,59,60,61,62,63,64,65,66,67] but never validate them in OSA. If we assume that the fixed AVR model was valid in OSA, we could expect that arm_OD highly correlates with finger_OD considering their mathematical relationship, e.g., a 1% decrease in fingertip arterial SpO2 may correspond to a 0.25% or 0.3% decrease in arm StO2 because only *a* percent (25% or 30%) of the 1% SpO2 desaturation can contribute to StO2 desaturation according to Formula (1). Thus, similar to the results of finger_OD in response to CPAP titration shown in Table 2, arm_OD should also decrease stepwise with increasing CPAP pressures. However, the lack of correlation between the changes in finger_OD and arm_OD and the missing association between CPAP pressures and arm_OD contradict this assumption, suggesting the fixed AVR model is unlikely to be valid in OSA. In fact, an OSA event is actually associated with increased vasoconstriction in peripheral limb arteries and arterioles [68,69], which suggests the AVR in the arm muscle is hardly constant during OSA. Most likely, coefficient *a* decreases while coefficient *b* increases in OSA because: (1) *a* + *b* = 1, and thus an increase in one coefficient must be associated with a decrease in the other, and arterial vessels have a stronger capacity to constrict than venous vessels; and (2) it is known that blood pressure, HR, left ventricular stroke volume and cardiac preload all decrease during apnea/hypopnea events [51,68,70,71,72], indicating more blood may be held in the venous vascular bed. Therefore, the contribution of venous blood to StO2 increases while arterial blood contribution decreases during OSA events, leading to a reduction in the sensitivity of StO2 in response to SpO2 changes. This interpretation also fits our results that arm_OD has a negative association with the mean HR during the events (Table 1), i.e., a higher HR may indicate less vasoconstriction (i.e., relative larger *a*) and relatively more arterial blood supply to the muscle tissues; ergo arm_OD is smaller.

To the best of our knowledge, NIRS has not been used to measure the peripheral hemodynamics (i.e., muscular hemodynamics) in OSA. Our results of no correlation between the amplitudes of finger_OD and arm_OD and the aforementioned increased venous contribution to StO2 during OSA events indicate that our arm_OD measured by NIRS is likely to mainly represent the OD in venous blood. Our results (Table 1 and Table 2) thus provide new insights into the changes in peripheral oxygen desaturation in OSA events during CPAP that (1) the arterial but not venous desaturation is more sensitive to changes in CPAP pressure; (2) arterial but not venous desaturation depends on the types of events (i.e., apnea causes larger desaturation than hypopnea) and sleep stages (light sleep causes larger desaturation than others); and (3) longer events cause larger desaturations in both arterial and venous blood as indicated by longer hypoxia that causes stronger oxygen extraction from both arterial and venous vascular bed.

In our recent study using the same database, we reported poor agreement (analyzed by Bland–Altman plots) between arm_OD and finger_OD in sleep hypopneas, and thus the reliability of the AHI measured by StO2 desaturation using wearable or portable optical sensors based on the NIRS technique is questionable [32]. This conclusion could be extended to wearable optical sensors based on an R-PPG technique such as wrist smartwatches because they face the same problem of venous blood influence as NIRS [32]. A recent study tested the accuracy of a wrist R-PPG smartwatch in measuring SpO2 when SaO2 measured from blood samples with a co-oximeter changed from 100% to 70% [73]. In that study, the Bland–Altman plot gave broad 95% lower (i.e., approximate −4%) and upper (i.e., approximate 6–7%) limits of agreement between smartwatch and co-oximeter measurements, which are similar to those reported in our study [32]. The authors also showed that the SpO2 measurement error of their smartwatch is 3% [73]. This accuracy is still too poor to measure sleep hypopneas because hypopnea is defined as ≥ 3%OD. Our results do not support the hypothesis that CPAP titration effectiveness may be assessed by measuring peripheral StO2 desaturation. This conclusion probably can apply to SpO2 measured by smartwatches too.

Our study has several limitations. First, our patients may only represent male patients with severe OSA because although their age (Shapiro–Wilk normality test: *p*-value = 0.45) and BMI (Shapiro–Wilk normality test: *p*-value = 0.66) follow normal distribution, only three females were included. Whether our conclusions can be generated to females and patients with moderate OSA needs further studies. Second, correlation and association are not causality. Although we controlled multiple covariates (e.g., HR, sleep stages) in our LMM models, the causal relationships between CPAP pressures and changes in oxygen saturations in fingertip and arm muscle need further studies, e.g., studies with randomized CPAP pressures and/or multi-parameter (e.g., blood flow, endothelium function) measurements in addition to oxygen saturation. The causality analysis can essentially provide new insights into the hemodynamic regulations and consequences of CPAP therapy in OSA [74,75].

## 5. Conclusions

Although the recent boom of wearable optical sensors, such as smartwatches and armbands, offers a possibility of assessing OSA and multiple-night CPAP titrations at home, our negative results should warn the general public and sleep researchers/clinicians to be cautious with these wearable devices until those products are clinically and experimentally validated. More sophisticated algorithms such as machine learning are probably needed to derive some peripheral parameters that can correctly measure SpO2 using wearable devices. Our results also suggest that the muscular StO2 desaturation measured by NIRS may primarily represent venous desaturation in OSA. We suggest that more studies including the gold-standard invasive measurements of SaO2 and SvO2 together with simultaneous non-invasive NIRS measurement during OSA events are needed to further test the robustness and reliability of NIRS as a non-invasive tool in measuring SvO2 in OSA.

## Figures and Tables

**Table 1:** The results of the linear mixed-effects model predicting the degree of oxygen desaturation measured at arm muscle.

	Estimate (10^−2^)	95% CI (10^−2^)	t-Value	*p*-Value
Duration of event	2.33	[2.00, 2.66]	13.89	<0.0001
Mean HR within events	−1.49	[−2.14, −0.84]	−4.50	<0.0001
Per-hour AHI	0.12	[0.025, 0.22]	2.47	0.014

CI: Confidence interval. HR: Heart rate. Per-hour AHI is the number of apnea/hypopnea events divided by the sleep duration under each CPAP pressure per hour.

**Table 2:** The results of the linear mixed-effects model predicting the degree of oxygen desaturation measured at fingertip.

	Estimate (10^−2^)	95% CI (10^−2^)	t-Value	*p*-Value
Duration of event	7.83	[7.01, 8.66]	18.68	<0.0001
CPAP pressures	−12.12	[−13.93, −10.31]	−13.10	<0.0001
Hypopnea–Apnea	−115.6	[−134.9, −96.3]	−11.71	<0.0001
Per-hour AHI	1.42	[1.14, 1.70]	9.87	<0.0001
Sleep stages				
Deep sleep–light sleep	−68.82	[−89.56, −48.08]	−6.51	<0.0001
REM sleep–light sleep	−56.41	[−82.13, −30.69]	−4.30	<0.0001
AHI of diagnostic night	1.93	[0.35, 3.50]	2.39	0.024

CI: Confidence interval. CPAP: Continuous positive airway pressure. REM: Rapid eye movement sleep. Per-hour AHI is the number of apnea/hypopnea events divided by the sleep duration under each CPAP pressure per hour. Hypopnea–Apnea means the change in apnea is the reference for the change in hypopnea in this model, i.e., the changes in hypopnea minus the changes in apnea. Non-rapid eye movement light sleep (stage N1 and N2) is the reference for deep sleep (stage N3) and REM sleep.

## Data Availability

The raw data supporting the conclusions of this article are available from the corresponding author upon reasonable request.
